# All-age whole mount *in situ* hybridization to reveal larval and juvenile expression patterns in zebrafish

**DOI:** 10.1371/journal.pone.0237167

**Published:** 2020-08-07

**Authors:** Franz Vauti, Luisa A. Stegemann, Viktoria Vögele, Reinhard W. Köster

**Affiliations:** Division of Cellular & Molecular Neurobiology, Zoological Institute, Technische Universität Braunschweig, Braunschweig, Germany; National University of Singapore, SINGAPORE

## Abstract

The zebrafish *Danio rerio* is a valuable and common model for scientists in the fields of genetics and developmental biology. Since zebrafish are also amenable to genetic manipulation, modelling of human diseases or behavioral experiments have moved into the focus of zebrafish research. Consequently, gene expression data beyond embryonic and larval stages become more important, yet there is a dramatic knowledge gap of gene expression beyond day four of development. Like in other model organisms, the visualization of spatial and temporal gene expression by whole mount *in situ* hybridization (ISH) becomes increasingly difficult when zebrafish embryos develop further and hence the growing tissues become dense and less permeable. Here we introduce a modified method for whole mount ISH, which overcomes these penetration and detection problem. The method is an all in one solution that enables the detection and visualization of gene expression patterns up to the late larval stage in a 3D manner without the need for tissue sectioning and offers a valuable extension for whole mount ISH by immunohistochemistry in the zebrafish field.

## Introduction

Whole mount *in situ* hybridization (WISH) detection of expressed mRNAs is an invaluable tool for research and diagnostics and a powerful application to study the cell- and tissue-specific expression of many genes in an organism. Different ISH protocols have been developed [1–3] and adapted for species that are frequently used in developmental biology and genetics, such as fruit flies, chick, frog, worms, mice and fish. The visualization of the spatial distribution of gene transcripts in single cells, tissues and embryos provides important information regarding developmental processes, health and disease [4–6].

The zebrafish *Danio rerio* has emerged as a useful model system for vertebrates because of a susceptibility to many tractable techniques for analysis of gene expression and function in whole mounts. The embryos are optically clear up to day 3 post fertilization (dpf). Based on the excellent WISH protocol for zebrafish [7], many patterns of expressed genes have been published in high-resolution in embryos and early larvae [8,9]. Most of the published gene expression patterns in zebrafish are available online at the Zebrafish Information Network (ZFIN, https://zfin.org/action/expression/search/). When querying the database (anatomy terms: including substructures; assay: mRNA *in situ* hybridization), 11620 genes are currently listed with expression patterns from the zygote to the larval protruding mouth stage (60–72 hours post fertilization, hpf) but only 2815 genes with expression data from larval day 4–6 (96–144 hpf). In later developmental stages most gene expression patterns are indicated for superficial body structures, isolated organs or tissue sections, rather than whole mounts. As few as 315 genes with expression pattern results are listed for larval stages between 7–13 (dpf), 122 genes with expression for larval stages between 14–20 dpf and 115 genes for expression pattern between days 21–29. In sum, 87% of gene expression patterns in zebrafish are published for the first 6 days and only 13% for the second to fourth week of development. These database entries in ZFIN, although the numbers just give a current snapshot, clearly indicate that the vast majority of the analyzed gene expression patterns is shown for embryos and early larvae. There is a clear lack of gene expression data in whole mounts at later larval stages, which represents a significant knowledge gap.

Many crucial steps affect the performance of *in situ* hybridizations. In general, the fixation and integrity of tissues, inactivation of nucleases, specificities and quantity of probes, hybridization and stringency of washing conditions and enzymatic or fluorescent activities of the hapten bound antibodies play an important role for *in situ* hybridizations.

Yet, the limiting step in whole mounts is the decreasing penetrance of probes to gain access to its cellular target molecules, when embryos grow beyond a critical size at late developmental stages. Then, the laborious sectioning of embryos or organs, followed by *in situ* hybridizations, is the method of choice to visualize gene expression patterns in larger and non-transparent tissues. Since current research in the zebrafish field is extended towards gene function analysis, disease modelling and/or behavioral analysis at later larval stages and in adults, a 3D reconstruction of gene expression patterns by whole mount ISH in older larval stages is a clear requirement in the field.

Here, we report a combined whole mount ISH protocol with substantial advance of existing techniques that allows a detection of gene expression in early embryos and concomitantly without any modification in late larval up to juvenile stages of the zebrafish. This protocol therefore allows for closing the gap of knowledge about WISH expression data between early larval and juvenile stages and could have a significant impact on research in developmental biology for the zebrafish community and likely other model organisms.

## Materials and methods

### Ethics statement

All zebrafish lines are housed in our fish facility according to the local animal welfare standards (Tierschutzgesetz §11, Abs. 1, Nr. 1) and in accordance with European Union animal welfare guidelines and legal regulations (EU-Directive 201_63). The facility is under the supervision of the local representative (Ordnungsamt Braunschweig) of the animal welfare agency (Permit # AZ 32.5./325-1-5-6-1). Zebrafish lines are maintained under constant 14 hours light/ 10 hours dark cycles at 28° C and treated according to the standard protocols [10] and to the Declaration of Helsinki for animal use and care. No animal experiments were performed on living animals. Embryos and larvae were anesthetized and sacrificed according to protocols approved by the Niedersächsisches Landesamt für Verbraucherschutz und Lebensmittelsicherheit (Permit # Az: 33.19-42502-05-16A070) using Tricaine as anesthetic following the protocol stated in [10].

### Embryo and larvae treatment

Eggs were collected after zebrafish mating in the morning and incubated in fish water in a humidified incubator at 28°C. Debris and unfertilized eggs were removed and the water was replaced by 30% Danieau-medium in the evening. To suppress residual pigmentation in embryos of the *brass* line, PTU was added after gastrulation (about 10 hours post fertilization). 30% Danieau/PTU medium was exchanged daily until embryos or larvae were harvested. Embryos and larvae from the *casper* line were treated the same, except that PTU was omitted from the 30% Danieau medium at any time. For details see the S1 File.

### Cloning and constructs

For cloning of the zebrafish *actn3*a construct, total RNA was isolated from an adult zebrafish using peqGOLD RNAPure™ (peqLab). 2μg of total RNA was reverse transcribed with oligo(dT) primers, a dNTP mix and SuperScript™ III Reverse Transcriptase (Thermo Fisher Scientific) for 1 hour at 50°C. The zebrafish *actn3a* partial cDNA was amplified using the following primers, to amplify the region between nucleotides 1725 to 2713 of the transcript (total length: 3209 nucleotides, NM_131522): forward primer: 5´-GGCAGCACCCTTCAACAACTGG-3´, reverse primer 5´-GTTCCCTTCTCAGCTCCTCCACTG-3´. The RT-PCR product (989 bp) was isolated (QIAGEN Gel Extraction Kit) and the purified fragment was cloned into the pGEM®-T Easy vector. The cloning of the *atxn1b* construct followed the same protocol. A partial sequence of the zebrafish *atxn1b* transcript (XM_005158159: *atxn1b* transcript variant X1, 6385 nt mRNA) was amplified using the forward primer 5´-CCAACAATGCAGGCCCATCACATAG-3´ and the reverse primer 5´-CAGACATCGCCCACAGAGAGTTTG-3´. The RT-PCR product (1849 bp) was isolated and cloned into the pGEM®-T Easy vector. The plasmids used for the synthesis of the other probes were kindly provided by A. McMahon (pB*Shh*), G. Hauptmann (pBzf*shha*), M. A. Akimenko (pBzf*dlx2*), M. Mione (pBzf*eomes*a) and K. Namikawa (pSC-B*pvalb7*).

### Quality control of RNA probes

Successful synthesis of DIG-labeled RNA probes was verified by visualization after electrophoresis of about one twentieth of the synthesized RNA before (control 1) and after DNaseI treatment (control 2) on 1% (wt/vol) agarose gels in 1x TAE buffer for 30 min. A good probe synthesis was indicated by a 5–10 times higher amount of RNA compared to the template DNA (control 1), which was not visible after DNaseI treatment (control 2). In case of RNA bands of variable length appeared, RNA quality was further analyzed to exclude degradation. A small volume containing 400 ng of DIG-labeled RNA (control 3) was dried in a vacuum centrifuge and then resuspended in 17μl RNA-sample buffer. The probe was denatured at 60°C for 10 min and chilled on ice. After addition of the loading buffer, the denatured RNA probe was loaded onto a 1.2% agarose gel containing formaldehyde. A clearly visible single band with the expected length of synthesized RNA after electrophoresis in 1x RNA running buffer indicated high quality of the probe. For details see the S2 File.

### Whole mount *in situ* hybridizations

The standard whole mount *in situ* hybridization using digoxigenin-labeled probes was performed with mouse embryos as described previously [11]. The new protocol for whole mount *in situ* hybridization on zebrafish embryos and larvae used in this study is described and commented in detail in the S3 File.

### Imaging and equipment

Stained embryos and larvae were stored in 70% ethanol/H_2_0. For image recording the tissues were transferred onto a microscope slide, the ethanol was sucked off and immediately replaced by 300 μl of 90% glycerol/H_2_0. After equilibration in the drop of glycerol (a few minutes), the larvae were positioned for the desired focus plane under a stereomicroscope (Leica MZFLIII). The visualized gene expression pattern of the zebrafish embryos and larvae were recorded by the Nikon DS-Fi3 microscope camera system and the NIS-Elements D software.

### Tissue sectioning

Following whole mount *in situ* hybridization the stained larvae were fixed in 4% PFA/PBST for 20 min and incubated overnight in 30% sucrose/PBS. The larvae were transferred into plastic wells, embedded in Polyfreeze Tissue Freezing Medium-Clear (Polysciences Inc.) and frozen at -80°C overnight. The larvae were sectioned using a Leica CM3050 S cryostat. The cryosections (7μm) were transferred onto glass slides, dried at 37°C for one hour and stored at -20°C. For image recording, the sections were thawed and incubated in 1x PBS for 5 min prior to the image documentation by the Nikon DS-Fi3 microscope camera system and the NIS-Elements D software.

## Results

### Problem of tissue penetration

Detection of mRNA by whole-mount ISH includes a series of applications that involve the synthesis of a labeled nucleic acid probe, fixation and preparation of embryos or tissues, hybridization of the probe to the target, washing to remove unhybridized probe and finally the visualization of the probe. The most crucial issue in preparing whole mount ISH is the initial proteolytic step of the tissues. A proper permeabilization is a prerequisite for labeled RNA probes to penetrate the whole tissue to gain access to its cellular target mRNA. The age and size of developing embryos, larvae or organs limit the success of the practical feasibility.

Most common protocols favor the use of Proteinase K to permeabilize the tissue. However, the enzymatic activities of each new stock solution and the proper time of incubations always have to be determined empirically before use. In most protocols the concentration of Proteinase K is reported in a range between 1 to 50 μg/ml [12] and the duration of proteolysis ranges between 5 to 40 minutes, depending on the given room temperature or 37°C. However, extended proteolysis results in tissue damage.

**Fig 1:**
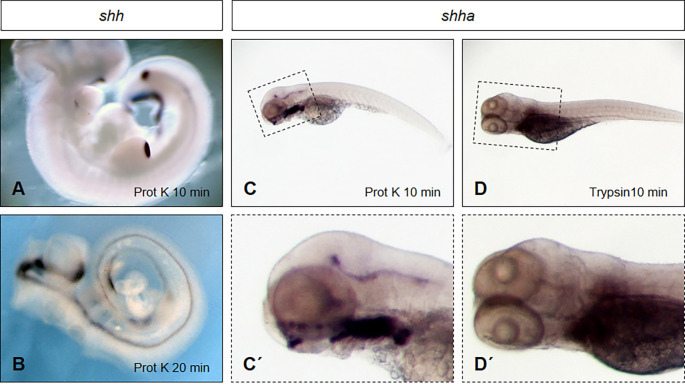
Protease treatment is critical for probe penetration. (A, B) Mouse embryos (E 10.5) were treated with Proteinase K (10 μg/ml) for 10 minutes (A) or 20 minutes (B) prior to probe hybridization with mouse antisense *shh* probes. (A) Shorter incubation time results in a rather superficial staining pattern in the zone of polarizing (ZPA) activity in limbs and posterior parts of the notochord. (B) Prolonged Proteinase K treatment damages the embryo integrity with loss of tissue, whereas hybridization is improved in deeper layers after *in situ* hybridization. (C) Safe Proteinase K treatment (10 μg/ml) in 5 dpf old zebrafish larvae without tissue disintegration indicates a staining pattern after hybridization with zebrafish specific antisense *shha* probe in the anterior part of the early larva (enlarged picture in C´). No signals are detected in the posterior tissues. (D) Improving tissue permeability by Trypsin digestion (10 μg/ml) prior to hybridization with antisense zebrafish *shha* probe is insufficient to detect *shha* expression in the corresponding tissues (enlargement in D´). For staining, all embryos were incubated in BM purple overnight.

We have successfully performed whole mount *in situ* hybridizations in mouse embryos in the past. The critical proteolysis step is illustrated in Fig 1. Mouse embryos (E 10.5) were treated with Proteinase K (10 μg/ml) for 10 minutes (Fig 1A) or 20 minutes (Fig 1B) prior to *in situ* hybridizations using antisense mouse *shh* probes. A rather superficial expression pattern is detected in the zone of polarizing (ZPA) activity in limbs and posterior parts of the notochord. Doubling of the duration time of Proteinase K treatment results in tissue loss (Fig 1B), but then *shh* targets can be reached in deeper tissue layers after *in situ* hybridization. The results clearly confirm that the duration of proteolysis is critical for detection of target mRNAs in outer or deeper cell layers of tissues but reaches its limits upon tissue disintegration.

We used a standard *in situ* protocol [11] to target *shha* specific antisense probes in zebrafish. Incubation of zebrafish early larvae (5dpf) for 10 minutes in Proteinase K (10 μg/ml) without tissue damage indicates an expression of *shha* in the floor plate, diencephalon and pharyngeal arches (Fig 1C, enlarged in Fig 1C´). However, no signals are detected in more posterior regions of the floor plate along the body axis in early larvae at 5 dpf. In addition, attempts with the alternative protease Trypsin (10 μg/ml) using the same incubation time prior to the hybridization also failed to detect *shha* expression in the corresponding tissues (Fig 1D, enlarged in Fig 1D´). The results clearly indicate that proper tissue proteolysis and penetration of probes to their targets is the most critical step in performing whole mount *in situ* hybridizations and limits the reliability of the approach to the early phase of embryogenesis in zebrafish.

### Proteinase K replacement by acetone solution treatment significantly improves tissue penetration for targeting molecules

In order to follow gene expression patterns in the zebrafish beyond embryonic stages of 5 dpf, we tested a number of modifications of the *in situ* hybridization protocols [7,11]. However, a prolongation of both, the Proteinase K treatment and hybridization time, was not successful for later larval stages (see S1 Fig). Although a longer proteolytic step with Proteinase K improved somewhat the probe penetration during a longer incubation time (60 hours), this was always on expense to the tissue integrity. A Proteinase K treatment (10μg/ml) up to 30 min (S1D Fig), or 60 min (S1E Fig) resulted in fragile tissues of *casper* larvae (15 dpf). Although a postfixation step by 4% PFA/PBS (20 min) was included after Proteinase K treatment, the tissues disintegrated along the following steps in the protocol. As whole mount *in situ* hybridizations (WISH) in older staged zebrafish larvae would require even longer times of proteolytic Proteinase K treatment we concluded that enzymatic proteolysis is not an option for WISH studies in zebrafish larvae older than 5dpf and systematically searched for alternatives to improve probe penetration.

Inspired by a report by Nagaso and coworkers in 2001, we omitted proteolytic steps completely and started to use acetone for RNA detection instead [13] followed by including additional organic solvent treatments. Our final protocol, with a combined use of ethanol, methanol, xylol, acetone and detergents, published here (a detailed protocol is provided in the S3 File, can be applied for all embryonic stages including late larval and juvenile stages of the zebrafish up to four weeks post fertilization, the oldest stage currently tried by us. Principal modifications of the preparation steps of embryos to overcome the limitation of tissue permeability prior to the prehybridization were found to be most decisive, whereas the steps post hybridization follow largely the standard applications.

We exchanged several steps in the ISH protocol [11] and tested different conditions using organic solvents in regard to their penetration abilities for probes and concomitantly the maintenance of tissues integrities. We developed a modified protocol, which finally fulfilled both criteria. In brief: zebrafish larvae were stored as usual in 100% methanol at -20° C. In a first step, the methanol was replaced by ethanol and the larvae were incubated in a mixture (1:1) of ethanol/xylol, washed again in ethanol and rehydrated in a descending ethanol series with water (0.1% Tween). The next step was an incubation in 80% acetone/H_2_O at -20°C followed by a bleaching of tissues prior to the prehybridization (for details see S3 File). We identified both organic solvents, xylol and acetone, to be essential for proper tissue penetrations, however to a different extent. When using both, xylol and acetone, a strong expression of *actn3a* in the posterior trunk region of *brass* larvae is detected at an age of 10 dpf (S1F Fig) and 15 dpf (S1I Fig). A minor effect on gene expression pattern detection is seen, when either the xylol step (S1G Fig) or the acetone step (S1H Fig) are omitted in 10 dpf old larvae. However, for older stage larvae the acetone step is critical (15 dpf) (S1K Fig). This acetone step is superior to the xylol step, which has a less, but still distinguishable, positive effect on signal detection in the older larvae (S1J Fig). In addition, we proved that both solvents enable a probe penetration and signal detection in peripheral as well in deep tissues of larvae. This is proved by the detection of *ataxin1b* transcripts in the telencephalon at 12 dpf (S1L and S1N Fig) or more superficial in the cerebellum at 17 dpf (S1M and S1J Fig).

The modified whole mount ISH protocol presented here is an outcome of different trials, validating its usefulness for several differently expressed genes. For these, we used antisense probes of genes with known expression patterns to test, whether our protocol confirms these expression domains in early embryos and, more importantly, detects the expression of these genes even in tissues of the following late larval and juvenile stages. Probes were selected for target detection in distinct regions of embryonic development and different germ layers in anterior or posterior regions, as well as in superficial and deep tissue layers.

**Fig 2:**
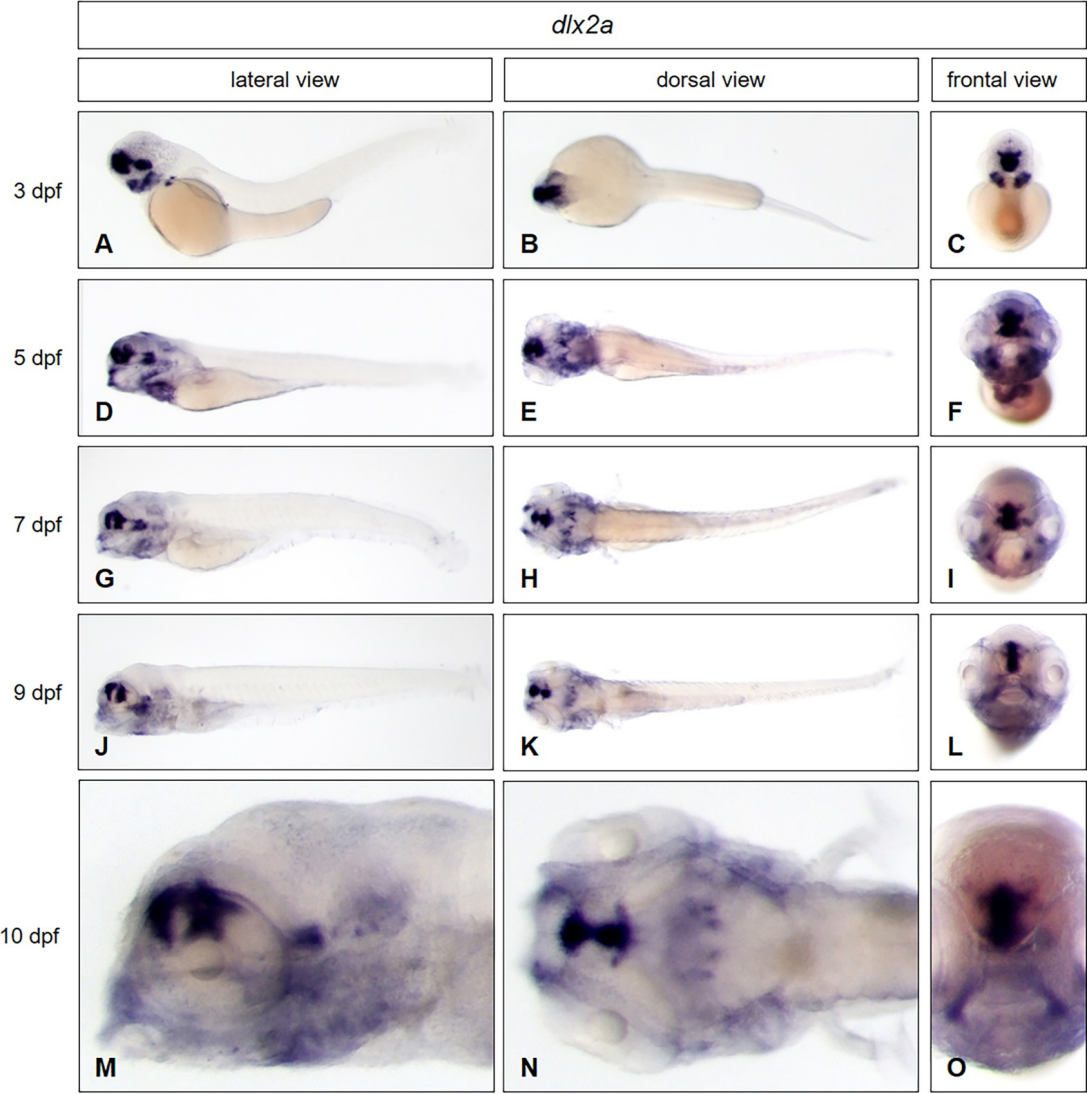
*Dlx2a e*xpression in zebrafish embryos and young larval stages. Whole mount *in situ* hybridization was performed in embryos and larvae at different developmental stages: (3 dpf: A—C; 5 dpf: D—F; 7 dpf: G—I; 9 dpf J–L; 10 dpf: M–O) and illustrated in lateral (A, D, G, J, M), dorsal (B, E, H, K, N) and frontal views (C, F, I, L, O). For staining, all embryos and larvae were incubated in BM purple for 22 hours.

**Fig 3:**
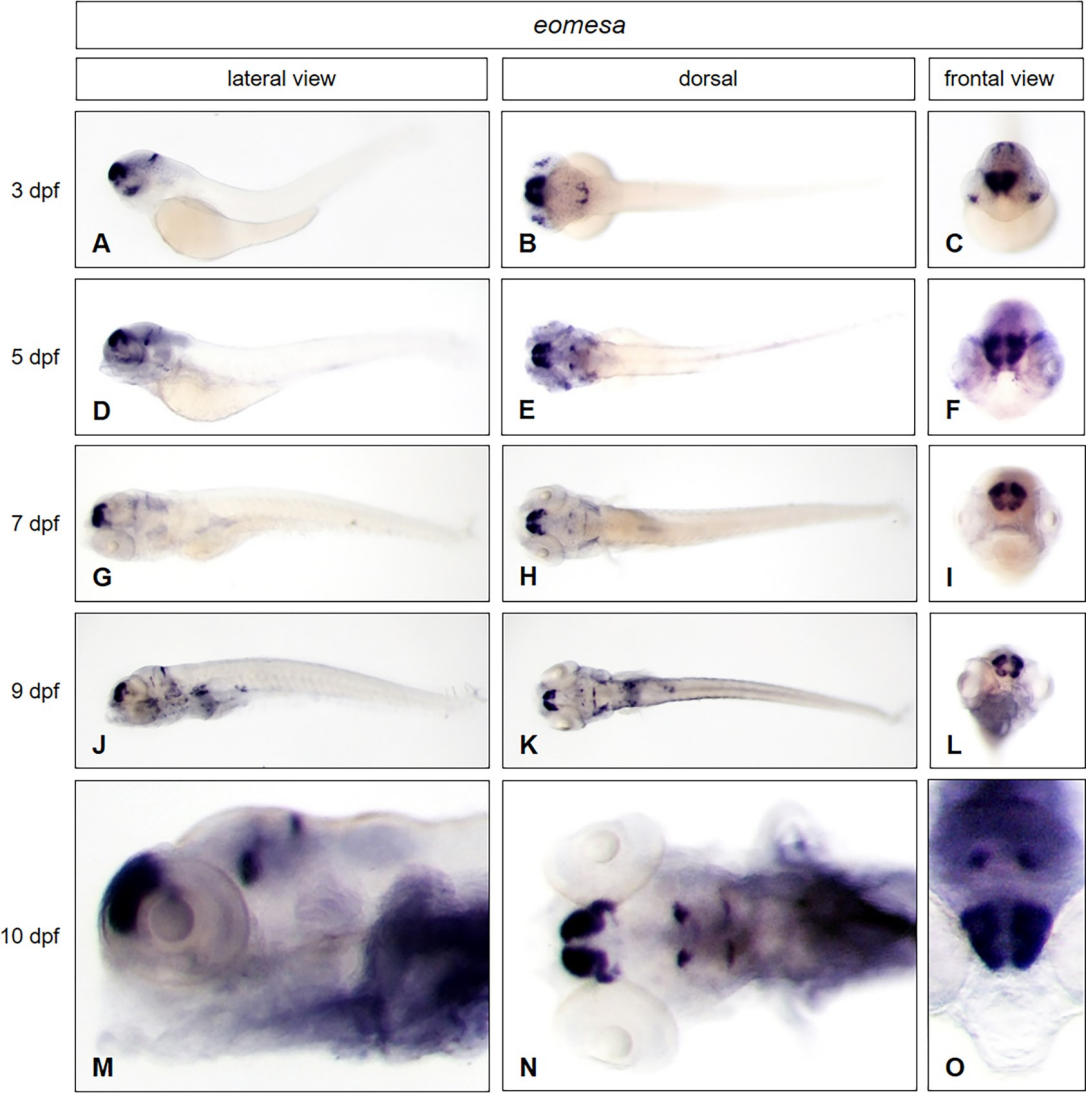
*Eomesa* expression in zebrafish embryos and young larval stages. Whole mount *in situ* hybridization was performed in embryos and larvae at different developmental stages (3 dpf: A—C; 5 dpf: D—F; 7 dpf: G—I; 9 dpf J–L; 10 dpf: M–O) and illustrated in lateral (A, D, G, J, M), dorsal (B, E, H, K, N) and frontal views (C, F, I, L, O). For staining, all embryos and larvae were incubated in BM purple for 22 hours.

First, we show that our new WISH protocol, without proteolytic steps is sufficient to confirm the distinct expression domains of *dlx2a* and *eomesa* in zebrafish embryos at stage of 3 dpf. The expression of *dlx2a* (Fig 2A–2C) is clearly visible in the telencephalon, diencephalon, hypothalamus and pharyngeal arch. The *eomesa* gene (Fig 3A–3C) is expressed in the dorsal telencephalon and diencephalon. The expression domains of both genes are confirmed by the published data in ZFIN and reported expression patterns [9]. In addition, the same pattern could also be visualized for *dlx2a* (Fig 2D–2O) as well as for *eomesa* (Fig 3D–3O) not only in whole mounts of later developmental stage at day 5, but even in larval stages at 7, 9 and 10 dpf. *Eomesa* for example is expressed in the Purkinje cell layer of the cerebellum at all indicated stages. These data clearly confirm that our new *in situ* protocol is able to detect the known expression patterns of *dlx2a* and *eomesa* in zebrafish embryos. Moreover, our protocol also works for whole mounts at larval stages from 5–10 dpf, when usually tissue permeabilities become restricted and sectioning is required before *in situ* hybridization. For optical transparency, all the embryos and the larvae used were treated with 1-phenyl 2-thiourea (PTU) during embryogenesis to inhibit pigmentation of the tissue.

### Expression of marker genes in different regions and developmental stages of zebrafish

**Fig 4:**
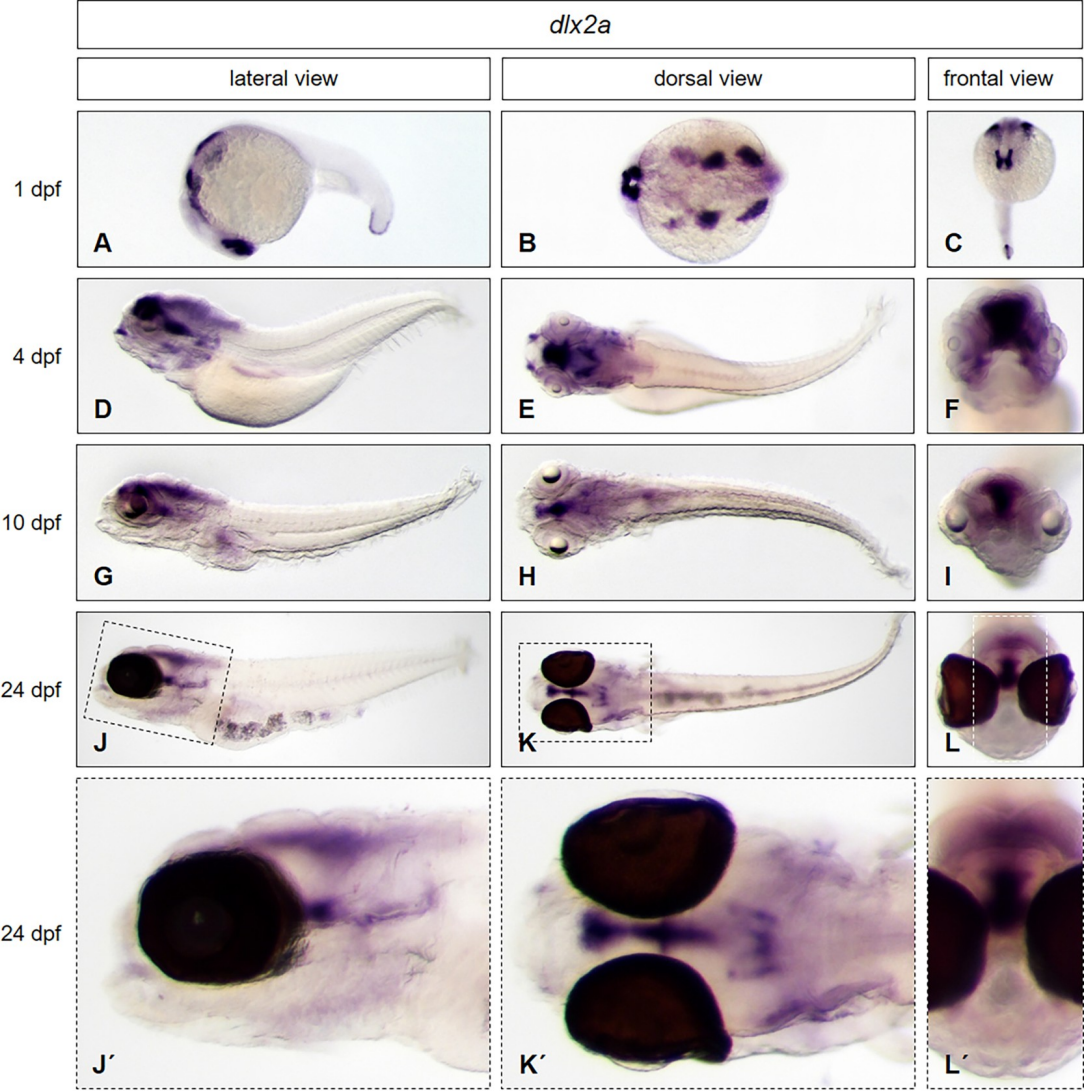
*Dlx2a* expression pattern from early embryonic to late larval stages is not affected by PTU treatment. Whole mount *in situ* hybridization was performed in embryos and larvae at different developmental stages (1 dpf: A—C; 4 dpf: D—F; 10 dpf: G—I; 24 dpf: J–L, enlarged in J´- L´) and illustrated in lateral (A, D, G, J, J´), dorsal (B, E, H, K, K´) and frontal views (C, F, I, L, L´). *Brass* wild type line embryos (A—I) and *casper* line larvae (J–L, J´- L´) have been used. Embryos at 4 dpf (D—F) and 10 dpf (G—I) were treated with PTU to suppress pigmentation, embryos at 1dpf (A—C) and 24 dpf (J–L, J´- L´) were untreated. For staining, all embryos and larvae were incubated in BM purple for 22 hours.

**Fig 5:**
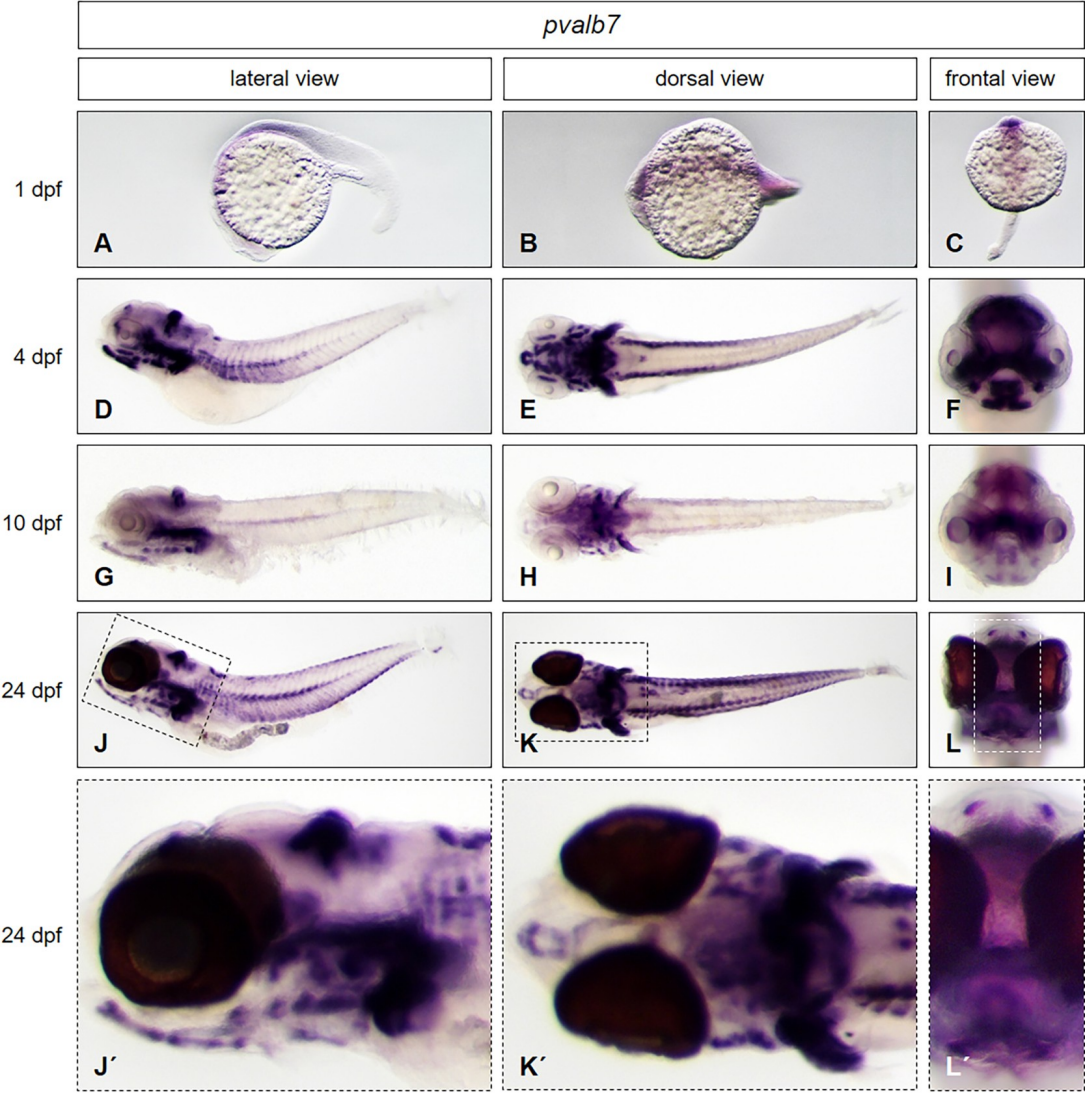
*Pvalb7* expression pattern from early embryonic to late larval stages. Whole mount *in situ* hybridization was performed in embryos and larvae at different developmental stages (1 dpf: A—C; 4 dpf: D—F; 10 dpf: G—I; 24 dpf J—L, enlarged in J´- L´) and illustrated in lateral (A, D, G, J, J´), dorsal (B, E, H, K, K´) and frontal views (C, F, I, L, L´). *Brass* wildtype line embryos (A—I) and *casper* line larvae (J–L, J´- L´) have been used. For staining, all embryos and larvae were incubated in BM purple for 22 hours.

**Fig 6:**
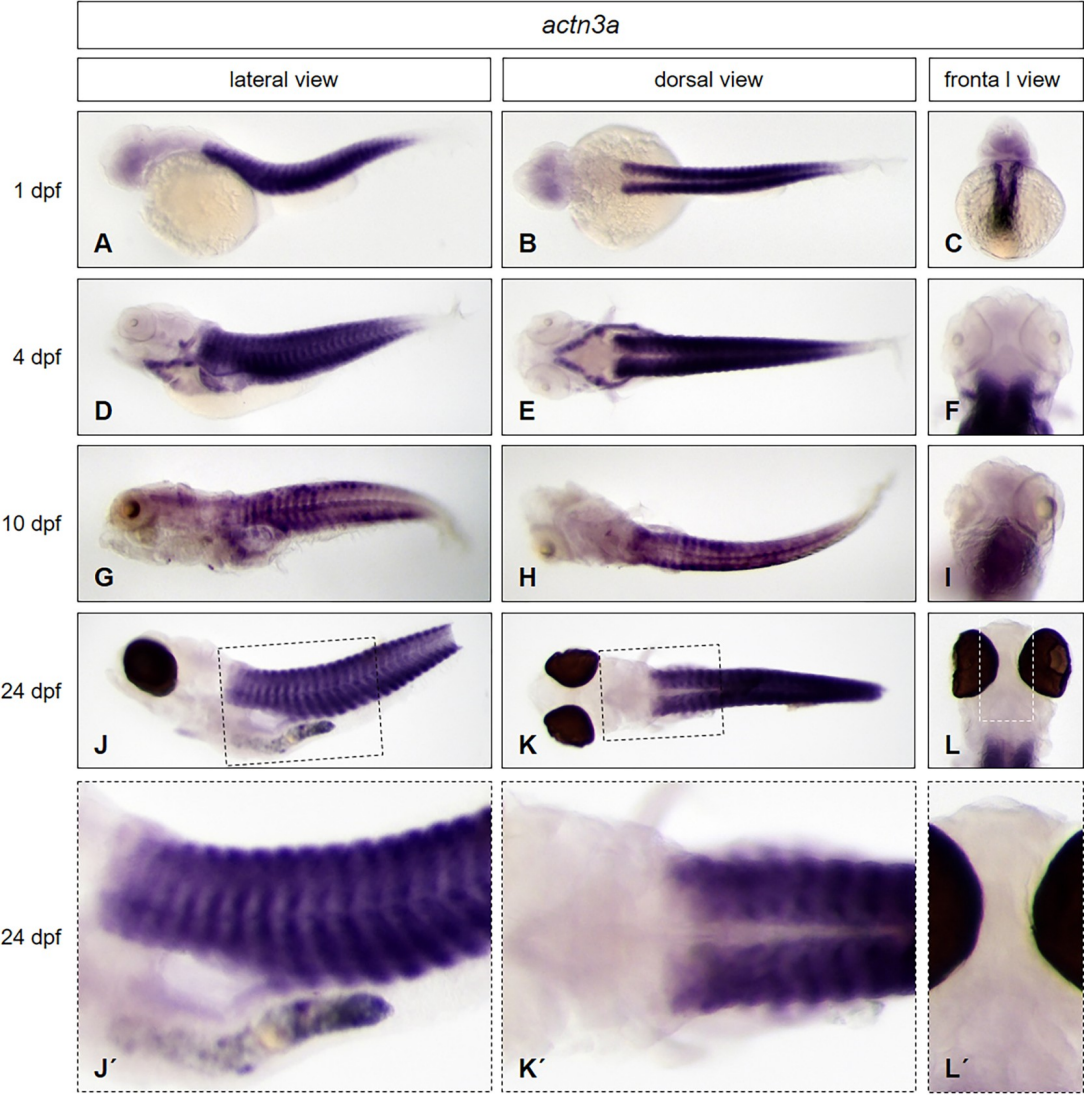
*Actn3a* expression pattern from early embryonic to late larval stages. Whole mount *in situ* hybridization was performed in embryos and larvae at different developmental stages (1 dpf: A—C; 4 dpf: D—F; 10 dpf: G—I; 24 dpf J—L, enlarged in J´- L´) and illustrated in lateral (A, D, G, J, J´), dorsal (B, E, H, K, K´) and frontal views (C, F, I, L, L´). *Brass* wildtype line embryos (A—I) and *casper* line larvae (J—L, J´- L´) have been used. For staining, all embryos and larvae were incubated in BM purple for 22 hours.

**Fig 7:**
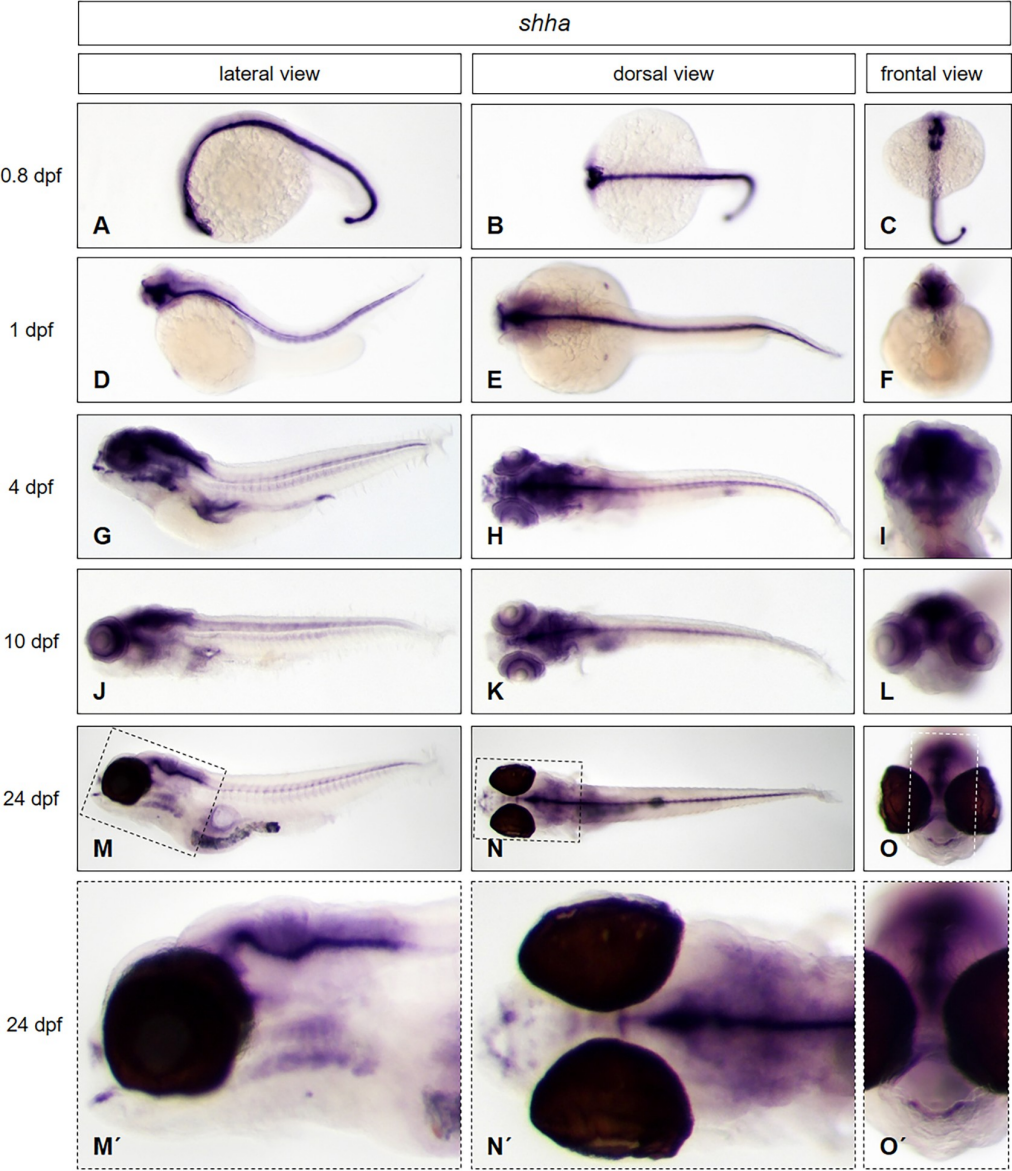
*Shha* expression pattern from early embryonic to late larval stages. Whole mount *in situ* hybridization was performed in embryos and larvae at different developmental stages: (0.8 dpf: A—C; 1 dpf: D—F; 4 dpf: G—I; 10 dpf: J—L; 24 dpf: M–O, enlarged in M´- O´) and illustrated in lateral (A, D, G, J, M, M´), dorsal (B, E, H, K, N, N´) and frontal views (C, F, I, L, O, O´). *Brass* wild type line embryos (A—L) and *casper* line larvae (M–O, M´- O´) have been used. For staining, all embryos and larvae were incubated in BM purple for 22 hours.

To test if our protocol is convenient for use at any developmental stage without any need of modification, we used *brass* wildtype embryos and larvae at three different developmental stages (1 dpf, 4 dpf *and* 10 dpf) and larvae from the *casper* line (24 dpf) together in one single tube to perform whole mount *in situ* hybridization. By using embryos and larvae of the transparent *casper* line, we further wanted to prove, that the expression pattern of the selected genes in larvae is not influenced by the presence of PTU, used above, which is normally used for inhibition of pigmentation. We applied antisense probes of genes that are expressed from the head to the trunk, in different morphological regions of the zebrafish: *distal-less homeobox gene 2a* (*dlx2a)*, *parvalbumin 7* (*pvalb7)*, *actinin alpha 3a (actn3a)* and *sonic hedgehog a* (*shha)*. The results are presented in Figs 4 to 7.

To confirm the expression pattern of *dlx2a*, observed in PTU treated embryos before (Fig 2), we used again PTU treated larvae (*brass*: 4 dpf and 10 dpf) as reference and untreated embryos at 1 dpf (*brass*) and larvae at 24 dpf (*casper*) and performed our ISH protocol. The results indicate the expected expression domains of *dlx2a* in ventral diencephalon, telencephalon/diencephalon ventricular zone, hypothalamus, the branchial arches and more posterior in the tail bud epidermis (1 dpf) (Fig 4A–4C). This expression pattern was also described previously [9]. The dominant expression domain in the telencephalon and diencephalon is pronounced at 4 dpf (Fig 4D–4F) that has been shown before [14] and is still obvious in larval stages at 10 dpf (Fig 4G–4I) and even at 24 dpf (Fig 4J–4L, and enlarged in Fig 4J´–4L´), which has not been shown before in whole mounts. Therefore, the new protocol enables the detection of transcripts in deep neuronal layers of the brain, in the ventral parts of the telencephalon and thalamus in late larval stages.

The expression of *pvalb7* is weak and rather diffuse in early embryos at 1dpf (Fig 5A–5C). Expression is detected in parts of anterior somites, dorsal spinal cord and cephalic floor plate [8]. At 4 dpf expression is detected in dorsal midbrain, hindbrain, in parts of the head, myotomes and muscles (Fig 5D–5F). A distinct expression of *pvalb7* is in the Purkinje cell layer of the cerebellum [15]. These domains are still present in larvae at 10 dpf (Fig 5G–5I) and even in late larvae at 24 dpf, with strong expression in muscles and the cerebellum (Fig 5J–5L, enlarged in Fig 5J´–5L´). In summary, by the use of the new protocol, the expression pattern of *pvalb7* is equivalent to published data in zebrafish at 4dpf and is continued to late larval stages at 10 and 24 dpf, which could not be shown before.

In many cases, gene expression patterns in the zebrafish are shown for anterior regions of the embryo, especially in the head region. To test the ability to detect target mRNA in structures of posterior embryonic regions like the trunk, we have chosen the *actn3a* gene as a marker for muscle tissue in zebrafish [8,16]. *Actn3a* is already strongly expressed at early embryonic stage of 1 dpf throughout the somites [8] and (Fig 6A–6C) and myotome at 4 dpf (Fig 6D–6F). Expression of *actn3a* is seen in the axial musculature and is also detected in cranial and pharyngeal muscles at 4 dpf. The restriction of *actn3a* expression persists to the muscle tissue in the posterior region of the body at 24 dpf (Fig 6J–6L, enlarged in Fig 6J´–6L´). No expression of *actn3a* is detected in the head region of this larval stage. Hence, using our new protocol confirms the expression domains of *actn3a* in cranial and pharyngeal muscles and axial musculature of embryos at day 4 of development and clearly shows that the expression becomes restricted to axial musculature only at later stages of development (24 dpf).

Finally, we tested a marker for deeper cellular layers in embryos and larvae to test if our protocol is able to reach targets of deeper tissue structures in aging larvae. We used *shha* as probe to target mRNA in the floor plate. *Shha* is expressed in the cranial primordium and in the spinal cord floor plate, adjacent to notochord [17]. We also show that the floor plate marker is strongly expressed in these structures at 20 hours post fertilization (hpf) (Fig 7A–7C) and 1 dpf (Fig 7D–7F). The expression exceeds to the anterior region of the forebrain and the anterior and posterior region of the hypothalamus at 4 dpf (Fig 7G–7I). The dominant expression domain in the spinal cord is clearly detectable by the use of our new method in the larval stage at 10 dpf (Fig 7J–7L) and even in juveniles at 24 dpf (Fig 7M–7O, enlarged in Fig 7M´–7O´).

In summary, the expression patterns of the selected genes *dlx2a*, *eomesa*, *pvalb7*, *actn3a* and *shha* prove that our new protocol for whole mount *in situ* hybridization is able to target mRNA molecules in superficial and deeper animal tissues from the early embryonic to the late larval stages throughout the body plan in the head as well as in the trunk. The new WISH protocol closes the gap in late larval and juvenile expression data in zebrafish and provides new insights into gene activities beyond stages that could not be used for gene expression analysis in whole mounts so far.

## Discussion

The main advantage of whole mount *in situ* hybridization (WISH) is that it is a quick and reliable method to determine the spatial distribution of specific mRNAs and the temporal gene expression in tissues. Techniques have been developed for embryos of different animal species that are large enough to be handled and still small enough to avoid problems with respect to the penetration of reagents. It is difficult to visualize complete gene expression patterns by whole mount ISH when the embryo grow larger and tissues mature. Tissue sections are subsequently required to elucidate the details and to discern the exact cellular distribution of transcripts of internal tissues, while tedious serial sectioning is required for 3D reconstructions of expression patterns.

In the mouse, the optimal size range for whole mount ISH lies between embryonic day 6–9 [18]. Only targets in superficial tissues are accessible to the probes and only a partial expression pattern can be expected at late developmental stages. Furthermore, probe trapping in body internal structures and cavities gets more pronounced in older stages resulting in significant background staining. In zebrafish, the most significant caveat of whole mount ISH is the poor penetration of RNA probes after day 2 of development [7]. Interestingly, although the average embryo size increases only moderately from embryonic to larval stages, the penetration of reagents for whole mount ISH becomes clearly restricted after day 5 of the zebrafish development. When hatching (48–72 hpf), the total body length of the zebrafish embryo is about 3.1 mm at long-pec stage (48 hpf) and 3.5 mm at larval protruding-mouth stage (72 hpf). The body length increases during larval development: day 4 (3.7 mm), day 5 (3.9 mm), day 6 (4.2 mm), days 7–13 (4.5 mm), days 14–20 (6.2 mm) and days 21–29 (7.8 mm) [19] and ZFIN: https://zfin.org/zf_info/zfbook/stages/index.html). Compared to the embryo size at 72 hpf, late stage larvae have doubled their body size. However, a simple prolongation of the incubation time in whole mount ISH does not solve the problem for reagent penetration, because tissue characteristics, including especially the skin, changes during larval development.

The zebrafish skin density is determined by a massive collagen layer [20]. In early embryos (24 to 72 hpf), the skin is composed of two strata and collagen fibrils appear first in an acellular subepidermal space at 24 hpf. Until 48 hpf, the collagen matrix is produced by the epidermis only. From 72 hpf to 5 dpf, a new collagen matrix is generated. This period interestingly coincides with the decreasing efficiency for targeting probes in whole mount ISH. The collagen fibrils self-organize into several lamellae from day 5–7 onwards and organize progressively into a plywood-like structure and the stroma has thickened from 5 dpf to 20 dpf. Both, the basal epidermal cells and the dermal cells bordering the deep region of the dermis are involved in the production of collagen from 72 hpf to 20–26 dpf. Then, fibroblasts of unknown origin progressively invade the acellular collagenous stroma and differentiate into the scale-forming cells. The cells of the epidermal basal layer cease the collagen synthesis, which is now produced by the invaded fibroblasts in the dermal stroma, but the cells of the dermal endothelium continue to produce collagen [20,21].We assume, that the thickening of the stroma by the growing collagen matrix from postembryonic day 5 onward limits the permeability of the larval skin.

In order to detect intracellular mRNAs, cells must first be fixed with cross-linking agents such as paraformaldehyde and afterwards permeabilized. Three general types of reagents are commonly used for permeabilization: organic solvents such as methanol, Proteinase K and detergents such as Triton X-100 and Tween-20. Proteinase K loosens the cell connectivity in tissues, the organic solvents dissolve lipids and leave holes in membranes making them permeable to antibodies. The organic solvents also can coagulate proteins and can be used to fix and permeabilize cells at the same time [22]. However, these three types of reagents alone are not sufficient to permeabilize zebrafish larval tissues. Our modified protocol introduced here uses ethanol, methanol, xylol and acetone as organic solvents for the preparation of larval tissues. The combinations of these solvents, together with Triton X-100 and Tween-20 as detergents, turned out to be efficient for the permeabilization of all embryonic and larval tissues tested. Even mRNA in cells of deeper tissues could be targeted in 24 dpf old larvae.

The visualization abilities of expressed genes also depend on the clarity of the tested tissue. In zebrafish, pigmentation of embryos and larvae can be inhibited by the treatment of 1-phenyl 2-thiourea (PTU) during embryogenesis. PTU inhibits melanogenesis by blocking all tyrosine-dependent reactions in the melanin synthesis [23]. PTU is usually added in standard concentration of 0.003% (200 μM) to water before the pigmentation initiates and embryos remain transparent as long PTU treatment is continued. However, PTU should be used with caution when studying zebrafish embryogenesis, as it is reported to alter the threshold of different signaling pathways important during craniofacial development [23,24]. In this respect we were concerned, if a prolonged PTU treatment of zebrafish embryos, from day 1 to day 10 post fertilization, affects the proper development of larvae at later stages. So far, most PTU treatments were terminated, when embryos have been harvested for microscopic analysis before day 5–6 of development. It was found that even 0.003% PTU in water altered the retinoic acid- and insulin-like growth factor (IGF)-regulation of neural crest and the mesodermal components of craniofacial development [24]. Recent reports describe that PTU also inhibits a proper development of the thyroid in zebrafish [25,26]. Our concern was that the new protocol is suitable for properly developed larvae and not only for aged ones with a retarded development caused by PTU. Therefore, the *casper* line late larval stages have been added to the experiments. It is important to note that *casper* larvae and the PTU treated larvae are not part of a comparative study but that the *casper* larvae serve to better prove that the WISH protocol is also working on larvae beyond day 10 of development. Beside retinal pigmentation, *casper* is a nearly complete transparent line [27] and no PTU treatment is required for whole mount ISH. In case, larval expression patterns in the eye are to be studied we recommend to use larvae from the transparent crystal line, which also lacks pigmentation of the pigmented retinal epithelium [28].

For each of the target genes selected, we could confirm the known expression patterns in 1 dpf old embryos (without PTU treatment) and in 4 and 10 dpf old larvae (PTU treated) from the *brass* line and moreover in 24 dpf old *casper* larvae (without PTU treatment). The expression domains are visualized in superficial and deeper tissues simultaneously without the use of Proteinase K. In addition, the omission of the proteolytic step is not only beneficial, but also facilitates the procedure as no variations of enzyme activities, batch differences, incubation times, or variations in embryonic/larval sizes influence the outcome. When applying our protocol, superficial and deeper targets could be reached for hybridization without a loss of tissue integrity and gene expression domains.

Highest quality detection of mRNA molecules however can only be achieved at a significant expense of the required incubation times when using our protocol. While standard protocols usually provide clear results after 3 days, our method requires an 8 day procedure. This may be due to the increased body size of late larval and juvenile zebrafish compared to embryos. In total, the hands-on time in the experimental procedure is not dramatically prolonged, as we mostly recommend extended incubation times for reactions to obtain satisfactory results. These include the duration for prehybridization (4–6 hours), hybridization (60 hours) and washing steps after the antibody incubation (60 hours). The staining reactions for the gene expression patterns shown were terminated for all embryonic and larval stages after 22 hours, allowing the detection of most gene expressions domains in more saturated staining patterns. However, first specific signals appeared for *eomesa* after 3 hours, for *actn3a* and *pvalb7* after 4 hours and for *Shha* after 6 hours staining reaction in BM purple. For better visualization the staining reaction was continued overnight (in total 22 h). In case of the low expressed gene *atxn1b* the first reliable signals were detected after 22 hours and with saturation after 80 hours. When using our new ISH protocol, the signal to noise ratio is clear and the background staining remains negligible also after prolonged staining (2–4 days) which is necessary to detect low level gene expressions.

Apart from the time delay, our protocol offers the ability to acquire expression data in zebrafish larvae that was not detectable before in whole mounts. Some other features underline beneficial aspects of our protocol in the practical performance: First, the protocol is suitable simultaneously for early embryos and late larvae without any modification, only the final staining time may be terminated at different time points depending on the developmental age. Second, whole mount ISH can be performed in one and the same 2 ml tube for all procedures. Third, expression domains of weakly expressed genes can be detected even after prolonged staining reactions for several days. Background staining is greatly reduced or completely absent in tissues where the target mRNA is not expressed.

Our results show clearly that all the different markers that have been tested not only confirm the known expression profiles in embryos, but most importantly, extends additional information of expression pattern in 3D manner up to late larval stages. Given that increasing numbers of labs start to focus on juvenile morphology, histology, physiology and behavior this new WISH protocol provides the access to gene expression data in the entire organism. For example, social behavior in zebrafish only develops after the third week of development [29], involved gene expression data is required for these late stages to determine the underlying genetic mechanisms of neural circuitry function. Therefore, this protocol expands gene expression analysis beyond the embryonic stage up to juvenile stages.

## Supporting information

S1 File Embryo/larvae collection, chorion removal, fixation and storage. (DOCX)

S2 File Probe synthesis and quantity/quality control. (DOCX)

S3 File All in one whole mount *in situ* hybridization protocol for embryonic to juvenile stages in zebrafish. (PDF)

S1 Fig Essential changes of the ISH protocol to detect gene expression pattern in larval zebrafish. (PDF)
