# Supplementary material for: All-age whole mount in situ hybridization to reveal larval and juvenile expression patterns in zebrafish
Source: PLoS One. 2020 Aug 7;15(8):e0237167. doi: 10.1371/journal.pone.0237167 (PMC7413480; doi:10.1371/journal.pone.0237167)
Supplement: S1 File — (DOCX) [file pone.0237167.s001.docx]

**S1** File. **Embryo/larvae collection, chorion removal, fixation and storage.**

Adult female and male zebrafish are set up as pairs separated by a diagonal plastic divider in breeding tanks the evening before mating. The next morning, the separator becomes removed and the fish start mating. The collection and treatment of eggs is performed as follows.

1. Transfer the zebrafish back to their home tank and collect the eggs from the bottom of the tank by pouring them into a strainer.
2. Rinse the eggs with fresh fish water into 10 cm petri dishes (maximal 50 eggs/dish)
3. Clean the clutch under the stereomicroscope and remove debris and infertile eggs from the fertilized ones.
4. Incubate the embryos at 28°C in fish water until the desired developmental stage is reached. All embryos and larvae that are collected later than the gastrula stages (5.25 - 10.33 hours) have to be treated with 0.003% 1-phenyl-2-thiourea (P-7629 Sigma) in 30 % Danieau medium at the end of the gastrulation period to inhibit pigmentation. The PTU-medium needs to be replaced every day until the embryos or larvae are harvested. PTU inhibits the tyrosinase activity and the formation of the dark melanin in melanocytes and keeps the zebrafish transparent. Media replacement promotes synchronous development without delays.
5. Dechorionate embryos that are not hatched carefully with sharp forceps (Dumont No. 5) by gently making a tear in the chorion and turning it upside down so that the embryo falls out. Zebrafish embryos develop normally outside their chorions at 28°C.
6. Transfer PTU-treated and dechorionated embryos or hatched larvae at the appropriate developmental stage(s) into a strainer with 5 ml of 30% Danieau-medium into the first well of a 6-well plate.
7. When the collection is finished, take out the strainer with the embryos and larvae and transfer the strainer to the second well of the plate containing 5 ml of Tricaine solution for anesthesia.
8. Anesthetize the embryos and larvae until no movements are detectable.
9. Transfer the strainer to the third well containing ice cold 30 % Danieau-medium to sacrifice the anesthetized larvae.
10. Transfer the strainer to the fourth well containing 10 ml of icecold 4 % paraformaldehyde in PBST and transfer the 6-well plate into a sealed plastic box to avoid spreading of the toxic gas in the fixative.
11. Fix embryos overnight at 4°C.
12. The next day remove the embryos and larvae from the fixative and wash the strainer with the fixed tissues in the fifth well with 10 ml PBST for 5 min.
13. Continue with a second wash in 10 ml PBST in the sixth and last well.
14. Take a fresh 6-well plate and dehydrate the embryos and larvae gradually in wells with 5 ml of ascending ethanol solutions in 25 %, 50 %, 75 % ethanol/H_2_O (vol/vol) and 96 % absolute ethanol, and twice in 100 % methanol to dehydrate the tissues completely. Perform the dehydration steps in volumes of 5 ml of the corresponding solution at 4°C, each for 15 minutes.
15. Finally collect the embryos from the strainer and transfer them with methanol into 2 ml tubes. Replace the transferred volume by 1 ml of fresh methanol and store the embryos and larvae at -20°C at least over night or until use. Embryos and larvae can be kept frozen in 100 % methanol for several month.

**Notes:**

Continue to incubate the embryos at 28.5°C in Petri dishes containing fish water until the desired developmental stage is reached. If post-gastrulation stages are examined, the formation of melanin pigment needs to be prevented. This can be achieved by replacing regular fish water with 0.003 % 1-Phenyl-2-Thiourea (PTU) solution prepared in 30 % Danieau medium at the end of gastrulation. For larvae and embryos that develop beyond 24 hours post fertilization (hpf), a daily exchange of medium is necessary.

**Solutions:**

1. Danieau’s solution (300%)

| **Amount per 1 L** | **Component (stock)** | **Final concentration** |
| --- | --- | --- |
| 101.7 g | NaCl | 1740 mM |
| 1.56 g | KCl | 21 mM |
| 2.96 g | MgSO_4_•7H_2_O | 12 mM |
| 4.25 g | Ca(NO_3_)_2_ | 18 mM |
| 35.75 g | HEPES buffer | 150 mM |

Add water to 1 L and stir until dissolved. Store at 4°C. The pH is 7.6. Make a 30% working solution of embryo medium for zebrafish by diluting the 300% stock solution 1:10 before use.

1. PTU: 0.003 % 1-phenyl-2-thiourea in 30 % Danieau-H_2_0.
2. Tricaine: 16,8 mg 3-amino benzoic acid ethyl ester in 1000 ml 30% Danieau-H_2_0 for anesthesia.
