# Supplementary material for: All-age whole mount in situ hybridization to reveal larval and juvenile expression patterns in zebrafish
Source: PLoS One. 2020 Aug 7;15(8):e0237167. doi: 10.1371/journal.pone.0237167 (PMC7413480; doi:10.1371/journal.pone.0237167)
Supplement: S2 File — (DOCX) [file pone.0237167.s002.docx]

**S2** File. **Probe synthesis and quantity/quality control**

The synthesis of a sufficient amount of DIG-labeled RNA probes with high quality is an essential prerequisite for successful whole mount *in situ* hybridization (ISH). Care must be taken in each pipetting step, to avoid any contamination with RNases and degradation of probes. The protocol below includes 3 controls to validate a successfully synthesis of probes.

1. For the preparation of DIG-labeled RNA-probes the components listed below are added sequentially to a 1.5 ml microfuge tube. All the components are cooled on ice.

| **Volume** | **Component (stock)** | **Final** |
| --- | --- | --- |
| x μl | RNase-free H_2_O | add first, adjust to the total volume |
| x μl | linearized template-DNA | 2 µg |
| 4 μl | 5 x transcription buffer | 1 x |
| 2 μl | 0,1 M DTT | 10 mM |
| 2 μl | 10 x DIG RNA labeling mix | 1 x |
| 0,5 μl | RNase-inhibitor (40 U/μl) | 1 u/µl |
| 2 μl | T7-, SP6-, or T3-RNA-polymerase (20 u/µl) | 2 u/µl |
| 20 µl | total volume |  |

1. Mix the components and incubate the reaction for 2 hours at 37°C.
2. After probe synthesis remove 1 µl of the reaction mixture and store it on ice (for control 1).
3. Add 2 µl of RNase free DNase I (20 u/µl) to the reaction volume and incubate at 37°C for 20 - 30 min.
4. After digestion of the template DNA remove again 1 µl of the reaction mixture and store it on ice (for control 2).
5. Add 100 µl RNase-free water to the 20 µl reaction mixture, followed by 12 µl LiCl (8 M) and 300 µl absolute ethanol.
6. Mix well and incubate over night at -20°C.
7. The next day, spin the tube in a microfuge at 13000 rpm for 15 min to sediment the precipitated RNA-probes.
8. Carefully suck off the supernatant. Wash the RNA-probe pellet with 1 ml 70 % ethanol/ 30 % H_2_O. Spin tube for 5 min at 13000 rpm. Then suck off the supernatant
9. Repeat the washing step once again.
10. After removal of the supernatant spin down the microfuge briefly to collect the residual wash solution in the bottom. Suck off the remaining solution carefully.
11. Air dry the labelled RNA pellet.
12. Dissolve the synthesized DIG-labeled RNA probe in 30 µl cold RNase-free H_2_O and quantify the amount of RNA in an UV spectrophotometer (Nanodrop).
13. Remove a small volume from the probe containing 400 ng RNA probe (control 3). Transfer the aliquot to a fresh microfuge tube and dry the volume in a vacuum centrifuge.
14. Add 1 µl RNase-inhibitor (40 U/μl) to the RNA-probe solution, mix well and keep the probes frozen until use.
15. Analyze the quantity and quality of the synthesized probe as recommended below.

**Notes:**

The synthesis of DIG-labeled RNA probes (control 1), the proper linearization of template-DNA (control 2) and the quality of the purified probe (control 3) are visualized after gel-electrophoresis. Both 1 µl aliquots of control 1 and 2, are each supplemented with 9 µl H_2_O and 2 µl of loading dye and loaded into wells of a 1 % agarose gel in 1 x TAE buffer. After electrophoresis at 100 V for 30 - 60 min, the synthesized RNA is visualized after staining of the nucleic acids in the gel with ethidium bromide (or an alternative nontoxic fluorescent dye). As one twentieth of the reaction volume, the linear template DNA should be visible as a distinct band (100 ng) in lane of control 1 together with a band of synthesized RNA. In lane 2 (control 2), only the band of RNA should be present, because the template DNA is digested. In principle, a good synthesis of RNA is indicated by a five to tenfold higher amount of produced RNA and a significant stronger band compared to the template DNA. We highly recommend to verify the quality of synthesized RNA-probes after electrophoresis in a denaturing formaldehyde gel. For this, the small aliquot of the RNA solution (control 3) should be dried in a vacuum centrifuge (alternatively, the RNA can be precipitated and dried). In parallel, an aliquot of a RNA-ruler is treated the same. The dried RNA and the ruler are then each resuspended in 17 µl RNA-sample buffer, denatured at 60°C for 10 minutes, chilled on ice and supplemented with 3 µl of a loading dye. The RNA-suspension and the ruler are loaded into wells of a 1.2 % denaturing formaldehyde gel. Electrophoresis is performed at 100 V in 1x RNA-running buffer for 3 hours with buffer recirculation to avoid any pH gradient in the buffer between the electrodes. A distinct RNA-band with the expected length of *in vitro* synthesized transcripts should become visible under UV-illumination without any signs of degradation.

**Solutions:**

1. 1 x TAE electrophoresis buffer materials

| **Volume/amount** | **Component (stock)** | **Final** |
| --- | --- | --- |
| 4.84 g | Tris base [tris(hydroxymethyl)aminomethane] | 1 x |
| 1.14 ml | glacial acetic acid (17.4 M) | 1 x |
| 0.3 g | EDTA, disodium salt | 1 x |
| Add to 1000 ml | deionized water |  |

1. 1 x RNA sample buffer

| **Volume** | **Component (stock)** | **Final** |
| --- | --- | --- |
| 212 μl | RNase-free H_2_O | add first |
| 100 μl | 10 x RNA running buffer | 1 x |
| 175 μl | 37 % Formaldehyde solution | 6,4 % |
| 500 μl | 100 % Formamide solution | 50 % |
| 3 μl | 10 mg/ml Ethidium bromide | 30 ng/µl |
| 10 μl | 10 % SDS | 0.1 % |
| 1000 µl | total volume |  |

1. 10 x RNA running buffer pH 7.0

| **Volume** | **Component (stock)** | **Final** |
| --- | --- | --- |
| 200 ml | 1 M MOPS  (3-(N-Morpholino)propanesulfonic acid, 4-Morpholinepropanesulfonic acid) | 200 mM |
| 16.6 ml | 3 M Sodium acetate pH 5.2 | 50 mM |
| 10 ml | 0.5 M EDTA pH 8.0 | 5 mM |
| 773,4‬ ml | Double distilled H_2_O* | 1000 ml |

Add double distilled H_2_O to a volume of 800 ml, adjust the pH to 7.0 with 10 N NaOH, then adjust with volume to 1000 ml with double distilled H_2_O. Store in the dark. Dilute 10 x stock solution 1:10 with double-distilled H_2_O for use.
