# Supplementary material for: All-age whole mount in situ hybridization to reveal larval and juvenile expression patterns in zebrafish
Source: PLoS One. 2020 Aug 7;15(8):e0237167. doi: 10.1371/journal.pone.0237167 (PMC7413480; doi:10.1371/journal.pone.0237167)
Supplement: S3 File — (PDF) [file pone.0237167.s003.pdf]

S3 File. **All in one whole mount in situ hybridization protocol for embryonic to juvenile stages in zebrafish**

The following protocol was developed to perform whole mount *in situ* hybridizations (WISH) on zebrafish embryos and larval stages. Embryos and larvae of all stages can be treated jointly. The most important buffers and solutions for the performance are described. Only recipes for frequently used standard buffers are not listed.

The new protocol offers a one for all solution for all developmental stages from the early embryo to the late larva. All steps for one probe are performed on embryos and larvae in a single 2 ml tube and always with a volume of 1.6 ml per solution.

The following flow diagram shows the major differences of our new protocol to a standard *in situ* ISH protocol [11].

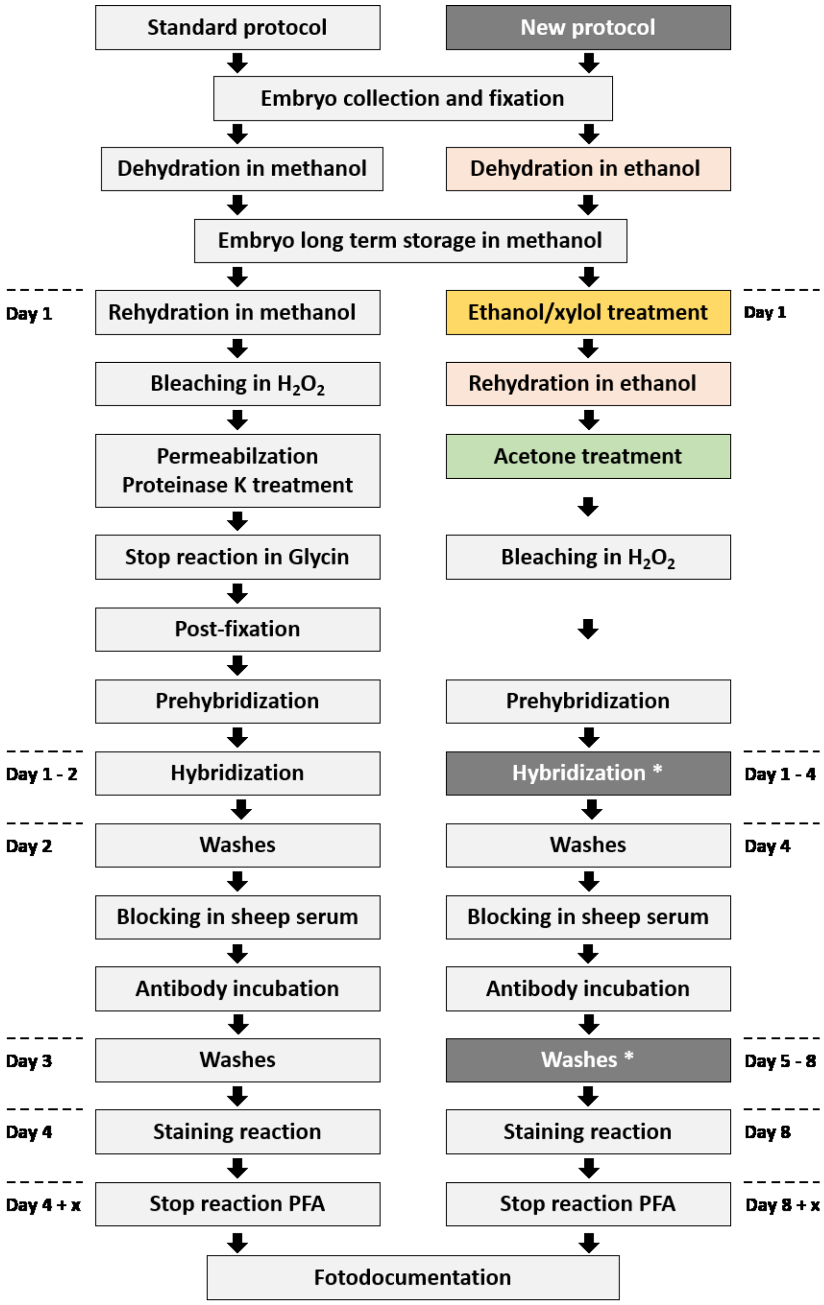

Flow diagram

The flow diagram shows the principal steps of a standard ISH protocol [11] on the left side (in grey). The experiments that differ in the new protocol are shown in colored boxes at the right side, unchanged steps although sometimes occurring in a different order remain in grey. The new steps include the mode of permeabilization of embryos and larvae and the prolonged times for the following hybridization and antibody washes (marked by asterisks). Although the new protocol takes longer, the experimental hands-on time is similar to the standard protocol as shown in the flow diagram.

Day 1

(1) **Permeabilization of embryos and larvae**

The permeabilization of embryos and larvae for whole mount ISH (WISH) described here differs from standard protocols and omits the proteolytic step by Proteinase K.

1. Collect the embryos and larvae that should be examined by one probe from the methanol storage and transfer them into one 2 ml tube.
2. Remove the methanol from the embryos and larvae by using a 1 ml pipette. The following washing and incubation steps are performed by mild movements of embryos and larvae at 20 to max. 50 rpm on a horizontal platform shaker unless otherwise stated.
3. Wash embryos and larvae in absolute ethanol for 5 min.
4. Wash in absolute ethanol a second time for 5 min.
5. Incubate embryos and larvae in an ethanol/xylol solution (1:1 vol/vol) for 1 hour.
6. Wash embryos and larvae in absolute ethanol for 5 min.
7. Wash in absolute ethanol a second time for 5 min.
8. Rehydrate embryos and larvae in descending ethanol-concentration (90 %, 75 %, 50 %, 25 % ethanol (vol/vol) in H_2O. Supplement H_2O with 0.1% Tween 20, to avoid a stickiness of the embryos to the tube. Each rehydration step is performed for 15 min.
9. Incubate in H_2O for 5 min.
10. Incubate a second time in H_2O for 5 min.
11. Permeabilize embryos and larvae in 80 % acetone/H_2O at -20°C for 30 min (no shaking at this step).
12. Wash embryos and larvae in PTwx for 5 min.
13. Wash embryos and larvae a second time in PTwx for 5 min.
14. Bleach embryos for 1 hour in 6 % H_2O_2 in PBST.
15. Wash embryos and larvae in PTwx for 5 min.
16. Wash embryos and larvae a second time in PTwx for 10 min.

Notes:

Embryos and larvae which will be hybridized with the same probe can be collected into the same single 2 ml tube. Depending on the developmental stages up to 50 embryos or larvae can be treated in the same tube for all processes. However, the tissue volume should not exceed more than maximal 10 % of the final washing solutions. All media exchanges are performed with 1 ml pipettes. After the incubations all solutions are sucked off. Care

must be taken to remove residual volumes. While the end of the tip glides from the wall to the bottom of the tube the embryos are shifted away carefully. The opening of the tip touches the bottom and the supernatant is slowly aspirated without shearing or damaging of any embryo. All incubations and washing steps that are done at room temperature can be performed with slight agitation (20 to max. 50 rpm) on a horizontal platform shaker. For embryos from zygote to early blastula stage, steps 3 to 7 and 9 to 11 can be omitted and only the rehydration (step 8), the following washes in PTwx (steps 12 and 13), the bleaching (step14) and washings (steps 15 and 16) are sufficient to continue with the protocol.

Solutions:

a) Absolute ethanol
b) Xylol
c) Acetone
d) 30 % Hydrogen peroxide
e) PBST: 1 x PBS/0.1 % Tween 20
f) PTwx: 1 x PBS/0.1 % Tween 20/0.1 % Triton

(2) Prehybridization and hybridization

Prepare the prehybridization solution and store it at 4°C until use. Then equilibrate the solution to room temperature (18 - 30°C) for a minimum of time until any precipitated detergents get dissolved and the solution becomes clear.

Prehybridization:

1. Remove the volume of PTwx from the preceding washing step completely.
2. Rinse embryos and larvae briefly in 0.5 ml fresh prehybridization solution.
3. Replace the volume again with 1.6 ml fresh prehybridization solution. Prehybridize the embryos and larvae for 4 - 6 hours without shaking at 65°C.

Hybridization:

4. Remove the solution from the preceding prehybridization period and replace it with

1.5 ml new prehybridization solution that has been preheated to 65° C before use.

5. Heat another 0.1 ml of the prehybridization solution to 85°C, add 80 ng of a RNA probe and incubate the probe for 3 minutes at 85°C. Then transfer the denatured probe solution (0.1 ml) directly into the freshly replaced prehybridzation solution (1.5 ml) with the embryos and larvae at 65°C.
6. Hybridize the embryos and larvae in 1.6 ml hybridization solution containing the corresponding probe (50 ng/ml) at 65°C without shaking for approximately 60 hours.

Notes:

The extended incubation time for the prehybridization (4 - 6 hours) and hybridization (60 hours) is critical and highly recommended in this protocol. For any probe we tested, the prolongation of incubation times favored the hybridization of the probes to their complementary mRNA in deeper tissues of late larval stages. A shorter hybridization step, typical overnight, might also be sufficient for more superficial targets or younger, embryonic stages and could be tested for individual probes. However, in case of the "one for all -protocol" provided here, the extended incubation time should be included, especially when older larvae are used. The probe concentration of all our probes tested was 50 ng per 1 ml hybridization solution. This is sufficient and probe concentration from 250 ng/ml up to 500 ng/ml did not improve the signal strength. The lower concentrations of probes (50 ng/ml) resulted in excellent hybridization signals after visualization and are therefore likely saturating.

Solutions:

a) Prehybridization solution

**Volume**	**Component (stock)**	**Final**
5 ml	100 % Formamide	50 %
2.5 ml	20 x SSC pH 4,5	5 x
50 µl	10 µg/µl Yeast-tRNA	50 µg/ml

1 ml	10% SDS	1%
10 µl	50 µg/ml Heparin	50 µg/µl
1,44 ml	DEPC-treated H_2O	
10 ml	total volume	

b) Hybridization solution: 80 ng of probe are denatured in 0.1 ml prehybridization solution at 85%°C for 3 minutes and then added to 1.5 ml prehybridization solution at 65°C. The final probe concentration is equivalent to 50 ng/ml.

Day 4

(3) <u>Posthybridization washings</u>

All washing steps at elevated temperatures are performed in prewarmed solutions in volumes of 1.6 ml without any shaking. Only washing steps at room temperature can be performed with slight agitation (20 to max. 50 rpm) on a horizontal platform shaker. We tested two different protocols (option A or option B) for post hybridization washings. Either can be used.

Washing option 1:

7. Remove the hybridization solution completely.
8. Wash 3 x 15 min in wash-solution 1 at 65°C.
9. Wash 2 x 5 min in a mixture (1:1) of wash-solution 1/wash-solution 2 (vol/vol) at 65 °C.
10. Wash 2 x 5 min in wash-solution 2 at room temperature.
11. Incubate embryos and larvae in 1.6 ml wash-solution 2 supplemented with 16 µl RNase (10 µg /µl) at 37°C for 60 min.
12. Wash 2 x 5 min in solution 2 at room temperature.
13. Wash 3 x 15 min in wash-solution 3 at 65°C.
14. Wash 2x 5 min in TBST at room temperature.

Alternative washing option 2:

- Remove the hybridization solution completely.
- Rinse embryos and larvae 1 x briefly in wash-solution A at 65°C.
- Wash 2 x 30 min in Wash-solution A at 65°C.
- Wash 2 x 30 min in Wash-solution B at 65°C.
- Wash 2 x 30 min in Wash-solution C at 65°C.
- Wash 1 x 30 min in Wash-solution D at 65°C

Notes:

For rather historical reasons we used wash option 1 which was obtained from the publication of Pizard and coworkers [11], as an alternate protocol for mouse and chicken. These washes use 50% formamide and 2 x SSC and an additional enzymatic digestion of unhybridized single strand RNA probes with RNase A. This wash option 1 has frequently been used in our lab for mouse embryos in the past, but it also works for zebrafish. More common in the zebrafish field are the protocols from Thisse [7]. This or similar protocols gradually wash embryos after hybridization with gradually decreasing parts of the pre-hybridization solution hybridization (100 %, 75 %, 50% 25 %) in 2 x SSC at temperatures between 60 - 70 °C. Highly stringent washings can also be achieved by a stepwise decrease of the salt concentrations in the washing buffers to remove unspecifically bound probes. This is the case in the wash option 2. We have used wash option 1 including the RNase treatment (Suppl Fig 1A) or wash option 2 with a highly stringent wash in 0.1x SSC (Suppl Fig 1B). Both options for the posthybrization washes provide highly stringent conditions for removal of unspecific probe bindings. If washings are performed with the prehybridization solution only for identical times and exchange frequencies like in wash option 2, the stringency condition is too low and the staining pattern shows a high background (Suppl Fig 1C). Therefore, either wash option 1 or wash option 2 can be used for our protocol.

Solutions for washing option 1:

a) Wash-solution 1:

**Volume**	**Compound (stock)**	**Final**
50 ml	100 % Formamide	50 %
10 ml	20 x SSC pH 4.5	2 x
10 ml	10 % SDS	1 %
30 ml	ddH_2O	
100 ml	Total volume	

b) Wash-solution 2:

**Volume**	**Compound (stock)**	**Final**
16.6 ml	3 M NaCl	0.5 M
1 ml	1 M Tris-HCl pH 7.5	10 mM
1 ml	10 % Tween 20	0.1 %
81,4 ml	ddH_2O	
100 ml	Total volume	

c) Wash-solution 3:

**Volume**	**Compound (stock)**	**Final**
50 ml	100 % Formamide	50 %
10 ml	20 x SSC pH 4.5	2 x
40 ml	ddH_2O	
100 ml	Total volume	

Solutions for washing option 2:

a) Wash-solution A:

Volume	Compound (stock)	Final
50 ml	100 % Formamide	50 %
10 ml	20 x SSC pH 7.0	2 x
1 ml	10 % Tween 20	0.1 %
39 ml	ddH_2O	
100 ml	Total volume	

b) Wash-solution B:

Volume	Compound (stock)	Final
10 ml	20 x SSC pH 7.0	2 x
1 ml	10 % Tween 20	0.1 %
89 ml	ddH_2O	
100 ml	Total volume	

c) Wash-solution C:

Volume	Compound (stock)	Final
1 ml	20 x SSC pH 7.0	0.2 x
1 ml	10 % Tween 20	0.1 %
98 ml	ddH_2O	
100 ml	Total volume	

d) Wash-solution D:

Volume	Compound (stock)	Final
0.5 ml	20 x SSC pH 7.0	0.2 x
1 ml	10% Tween 20	0.1%
98.5 ml	ddH_2O	
100 ml	Total volume	

(4) <u>Incubation with Alkaline phosphatase anti DIG antibody</u>

All following steps are performed with embryos and larvae protected from light. Transfer the tubes to a dark box after the medium exchange.

15. Incubate embryos and larvae in blocking solution (10 % sheep serum in TBST) for 4 hours with gentle agitation.
16. Preadsorbe 1.0 µl DIG- Anti-DIG-AP Fab fragments antibody (1:2000) in 2 ml 1 % sheep serum in TBST for 90 min.
17. Discard the blocking solution and replace it with the preadsorbed antibody solution.
18. Incubate the embryos and larvae with the antibody for 2 hours at room temperature and after that overnight at 4°C with slow agitation (20 to max. 50 rpm on horizontal orbital shaker).

Solutions:

a) Sheep serum
b) 10 x Tris buffered saline (TBS): 100 mM Tris-HCl, 1.5 M NaCl, pH 7.5

c) 1x TBST

Volume	Compound (stock)	Final
10 ml	10 x TBS pH 7.5	1 x
1 ml	10 % Tween 20	0.1 %
89 ml	ddH_2O	
100 ml	Total volume	

d) Blocking solution: 10 % sheep serum/TBST

e) Antibody solution: Dilute Anti-DIG-AP Fab fragments (Roche 1093274) 1:2000 in 2 % sheep serum (h.i.) in TBST.

Notes:

The embryos are incubated for at least 90 minutes in the blocking solution. This step saturates nonspecific protein binding sites for the antibody and can be extended to 3 - 4 hours, if background problems occur after staining.

Day 5

(5) <u>Removal of unspecifically bound and unbound antibodies</u>

The washing steps are performed in volumes of 1.6 ml under gentle agitation at room temperature in the dark.

19. Discard the antibody-solution.
20. Wash embryos and larvae 3 x 5 min in TBST.
21. Wash 8 x 30 - 60 min in TBST.
22. Wash 1 x 3 days in TBST at 4°C with gentle agitation.

Notes:

The last washing step is extended to 3 days. This prolongation step is perhaps not required when only embryos are used. However, we recommend this extended wash steps for larvae, or if background problems occur.

Day 8

(6) Staining, storage and photo documentation of embryos and larve

After the washing steps and removal of unspecifically bound antibodies with TBST solution, the embryos and larvae are prepared for the staining reaction.

23. Wash embryos and larvae 3 x 20 min with 1.6 ml TBST with gentle agitation.
24. Wash 3 x 20 min with 1.6 ml alkaline NTMT solution with gentle agitation.
25. Discard the alkaline NTMT solution, add 1 ml of BM purple to the embryos and larvae in the 2 ml microfuge tube. Do not agitate the tubes during the staining procedure to avoid any diffusion of the colored precipitate in the embryos and larvae. Incubate embryos and larvae with BM purple at room temperature in the dark until staining becomes visible. This can take less than one hour up to several days. Monitor periodically the staining pattern of embryos and larvae within the 2 ml tubes under an illuminated stereomicroscope until the desired staining intensity is reached. Avoid any overexposure to light and always keep the embryos in the dark between the observations.
26. When the desired staining intensity is reached, remove the staining solution completely and wash the embryos and larvae in stop solution (optional) or PBST and then gradually in 25 % - ethanol/ddH_2O, 50 %- ethanol/ddH2O and finally 70 %- ethanol/ddH2O for 15 min. The stained embryos and larvae can be kept and stored in 70 % ethanol/ddH_2O for several month at 4°C in the dark.
27. To record images remove one embryo or larva from the storage and place it centrally in the depression of a microscope slide. Aspirate the remaining 70 % ethanol/ddH_2O and add immediately a drop of 90 % glycerol/ddH_2O to the embryo or larva. It will last some minutes until the embryo becomes further dehydrated. After equilibration, the embryo is placed in the proper position and orientation. Images can be recorded with a camera set on a stereomicroscope.

Solutions:

a) 1 x NTMT

Volume	Compound (stock)	Final
1 ml	1 M Tris-HCl, pH 9.5	100 mM
0.5 ml	1 M $MgCl_2$	50 mM
	3 M NaCl	
0.1 ml	10 % Tween 20	0.1 %
8.4 ml	ddH_2O	
10 ml	Total volume	

b) BM-Purple: Roche

c) 90 % Glycerol/ddH_2O

d) 25 % -, 50 % - and 70 % Ethanol/ddH_2O

e) Stop solution (optional): 1x PBS, pH 5.5, 1 mM EDTA, 0.1 % Tween 20 (vol/vol)

Notes:

Dependent on the amount and tissue of the expressed target mRNA, the staining reaction should be controlled from time to time until an expression pattern becomes clearly visible. Staining can last from less than one hour to several days, depending on the developmental stage of embryos and larvae and the amount and local distribution of the targeted transcripts of expressed genes. Just in case of strongly expressed genes and a fast staining, the alkaline phosphatase reaction should be terminated by a stop solution. In most cases, the washing and dehydration procedure is sufficient to terminate the coloration.

We found that freshly prepared NBT and BCIP solution is less suitable for prolonged staining reactions in this new protocol, because background stain is more likely to become visible. Staining of embryos and larvae with 1 ml BM purple in locked 2 ml microfuge tubes in the dark is preferred. The solution keeps the yellow light color for days, it does

not turn pink and therefore background staining is mostly avoided. Because BM purple substrate solution is stabilized and presents superior signal-to-noise rations, it is recommended for use when long developing times (overnight to several days) are required [11].

Final note:

The one for all protocol introduced here provides reliable results of gene expression patterns in zebrafish embryos and larvae up to the juvenile stage. When using this protocol, improvements and simplifications will likely be made with further experiences and are welcome. This protocol offers a new option to fulfill the current need for the visualization of gene expression patterns in 3D pattern in whole mount larval stages, which is a clear benefit for the zebrafish field.
