# Supplementary material for: All-age whole mount in situ hybridization to reveal larval and juvenile expression patterns in zebrafish
Source: PLoS One. 2020 Aug 7;15(8):e0237167. doi: 10.1371/journal.pone.0237167 (PMC7413480; doi:10.1371/journal.pone.0237167)
Supplement: S1 Fig — (PDF) [file pone.0237167.s004.pdf]

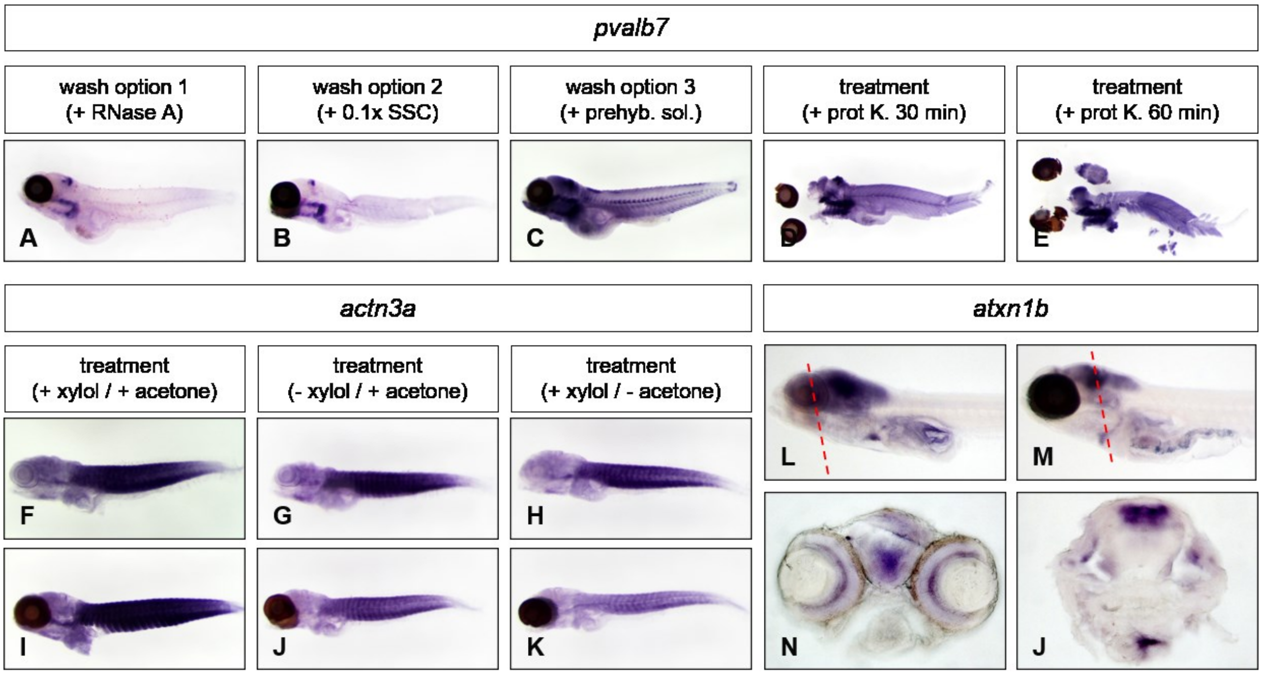

S1 Fig. Essential changes of the ISH protocol to detect gene expression pattern in larval zebrafish. The new protocol includes some major changes to standard ISH protocols. The post-hybridization washing steps include RNAse A treatment in the washing steps (A), a highly stringent final wash step with low salt concentration of 0.1 x SSC (B), or washings in pre-hybridization solution only (C). The prolonged incubation steps in the new protocol are less or not applicable for larval tissue integrity after pre-treatment with Prot. K for 30 min (D) or 60 min (E), when fragile tissues fall apart. Replacement of Prot K by a treatement with xylol and acetone (F, I), improves the hybridization signals as compared to acetone (G, J) or xylol (H, K) treatment only. This effect is less pronounced in younger larvae (10 dpf, F-H), however it is striking in older ones (15 dpf, I-K). Hybridization signals following our new WISH protocol (L, M) can be detected after tissue sectioning in deeper (telencephalon, N) and superficial brain tissues (cerebellum, J). The zebrafish larvae of the *casper*-line at the age of 15dpf (A–E and I-K) and 17 dpf (M, J) and *brass*-line at 10 dpf (F-H) and 12 dpf (L,N) were hybridized to antisense probes (50 ng/µl) of *parvalbumin 7* (*pvalb7*, A-E), *actinin 3a* (*actn3a,* F-K) and *ataxin-1b* (L-J). The larvae hybridized with *pvalb7* (Suppl Figs A-E) or *actn3a* (F-K) antisense probes were stained for 9 hours and the larvae with *atxn1b* antisense probes (Suppl Figs L-J) were stained for 80 hours in BM purple. The probe concentration was 50ng/ml hybridization solution.
